# Comparison of visceral fat mass measurement by dual-X-ray absorptiometry and magnetic resonance imaging in a multiethnic cohort: the Dallas Heart Study

**DOI:** 10.1038/nutd.2016.28

**Published:** 2016-07-18

**Authors:** I J Neeland, S M Grundy, X Li, B Adams-Huet, G L Vega

**Affiliations:** 1 Department of Internal Medicine, University of Texas Southwestern Medical Center, Dallas, TX, USA; 2 Dallas VA North Texas Health Care System, Dallas, TX, USA; 3 Center for Human Nutrition, University of Texas Southwestern Medical Center, Dallas, TX, USA; 4 Department of Clinical Sciences, University of Texas Southwestern Medical Center, Dallas, TX, USA; 5 Department of Clinical Nutrition, University of Texas Southwestern Medical Center, Dallas, TX, USA

## Abstract

**Background/Objectives:**

Visceral adipose tissue (VAT) mass, a risk factor for cardiometabolic complications of obesity, is usually measured by magnetic resonance imaging (MRI) but this method is not practical in a clinical setting. In contrast, measurement of VAT by dual-x-ray absorptiometry (DXA) appears to circumvent the limitations of MRI. In this study, we compared measurements of VAT mass by MRI and DXA in the large, multiethnic cohort of the Dallas Heart Study (DHS).

**Subjects/Methods:**

About 2689 DHS participants underwent paired measurement of VAT by MRI and DXA. Sex-stratified analyses were performed to evaluate the correlation and agreement between DXA and MRI. Model validation was performed using bootstrapping and inter-reader variability was assessed.

**Results:**

Mean age of the cohort was 44 years, with 55% female, 48% Black and 75% overweight/obese participants. Regression analysis showed a linear relationship between DXA and MRI with *R*^2^=0.82 (95% confidence interval (CI) 0.81–0.84) for females and *R*^2^=0.86 (95% CI 0.85–0.88) for males. Mean difference between methods was 0.01 kg for females and 0.09 kg for males. Bland–Altman analysis showed that DXA tended to modestly underestimate VAT compared with MRI at lower VAT levels and overestimate it compared with MRI at higher VAT levels. Results were consistent in analyses stratified by race, body mass index status, waist girth and body fat. Inter-individual reader correlation among 50 randomly selected scans was excellent (inter-class correlation coefficient=0.997).

**Conclusions:**

VAT mass quantification by DXA was both accurate and valid among a large, multiethnic cohort within a wide range of body fatness. Further studies including repeat assessments over time will help determine its long-term applicability.

## Introduction

Visceral adipose tissue (VAT) imparts risk for type 2 diabetes,^1^ hypertension^2^ and cardiovascular disease.^3, 4^ Moreover, reduction in VAT may potentially explain some of the improvements in cardiovascular disease risk seen with lifestyle,^5^ medical^6^ and surgical^7^ weight loss interventions. Although VAT can be accurately measured using dedicated techniques, such as magnetic resonance (MRI) and computerized tomography (CT) imaging, implementation of these modalities remain limited in both clinical practice and research investigation due to high cost, prolonged scan time (with MRI) and significant radiation exposure (with CT).

Estimation of VAT mass is now possible during the measurement of body composition using dual-x-ray absorptiometry (DXA). This method uses the differential attenuation of X-ray beams at two separate energies to calculate the soft tissue composition in a scanned region of interest and can be used to measure both whole-body and regional distribution of fat and lean mass.^8^ The effective radiation dose incurred during DXA scanning is relatively low^9^ (~1.5 mrem) and the scan time is of short duration compared with MRI or CT, making DXA a simpler, generally safer and faster technique than other modalities for serial measurements of body composition. Several studies that have examined the accuracy and precision of DXA methods to measure VAT mass report strong correlations (*r*>0.9) with expert manual or software-based measurements with CT imaging.^10, 11, 12, 13^ However, large, multiethnic populations with high proportions of both sexes have not been studied with DXA compared with MRI. Thus, we aimed to compare VAT mass quantified by paired MRI and DXA measurements in the Dallas Heart Study (DHS) population. We also sought to identify important sources of variability and limitations related to DXA VAT measurement in order to substantiate its use for future clinical and research applications.

## Methods

### Study population and variable definitions

The DHS is a multiethnic, probability-based, population cohort study of Dallas County adults with deliberate oversampling of African–Americans. Detailed methods of the DHS have been described previously.^14^ Briefly, between 2000 and 2002, 2693 subjects completed three DHS visits, including a detailed in-home survey, laboratory testing, and DXA and MRI imaging scans. Data on body composition and regional fat distribution measured by DXA and MRI have been published previously for this cohort.^15^ In the current study, the advanced version of the APEX software (Hologic Inc., Bedford, MA, USA) was employed to re-analyze the DXA images previously obtained and to generate VAT mass estimates to compare with previously published MRI VAT mass. For the present analyses, participants with irregular MRI or DXA data were excluded (*n*=4, one participant with negative VAT by MRI and three participants with exceedingly discordant DXA VAT data), yielding a final sample size of 2689. Age, sex, race/ethnicity, history of cardiovascular disease and smoking status were self-reported. Definitions for hypertension, diabetes, low high-density lipoprotein cholesterol and metabolic syndrome have been previously described using conventional clinical definitions.^16, 17^ Weight and height were measured using standard scales and body mass index (BMI) was calculated as weight in kilograms divided by height in meters squared; normal weight was defined as a BMI<25 kg m^−^^2^. Waist circumference was measured 1 cm above the iliac crest and hip circumference at the widest circumference of the buttocks at the area of the greater trochanters. High waist girth was defined as ⩾88 cm for females and ⩾102 cm for males.^17^ Participants provided written informed consent, and the protocol was approved by the Institutional Review Board of the University of Texas Southwestern Medical Center.

### MRI measurements

Subjects were imaged by a 1.5 Tesla MRI scanner (Intera, Philips Medical Systems, Best, The Netherlands) using a prospectively designed and validated method of fat mass prediction from a single MRI slice at the L2–L3 intervertebral level.^18^ Abdominal adipose tissue was separated into VAT (intraperitoneal+retroperitoneal fat) and subcutaneous adipose tissue (SAT) compartments by manually circumscribing contours using anatomical landmarks as detailed previously.^15^ Fat volumes were converted to mass using 0.9196 kg l^−1^ as the density of triglyceride in adipose tissue. Single-slice measurement of SAT and VAT fat mass at this intervertebral level has been shown to be highly concordant with total abdominal fat mass measured at all intervertebral levels (*R*^2^=0.85–0.96).^18^

### DXA measurements

Whole-body composition analysis was performed with a Discovery W DXA scanner (Hologic Inc.), as detailed previously^15^ and images were re-analyzed with APEX software version 13.4.2 for the present report. This software enables estimation of VAT mass at the L4–L5 region. The methodology for DXA VAT measurement has been described previously.^11^ Briefly, the lateral abdominal SAT seen in the DXA image was used to determine the anterior and posterior abdominal SAT, allowing VAT to be estimated from the total abdominal fat measured (total abdominal fat−total abdominal SAT=VAT, expressed in kg in the L4–L5 slice). As MRI VAT mass data are expressed as total mass (in kg) spanning the L1–L5 region, scaling factors were derived for the estimation of total VAT mass from L1–L5 measured by DXA as detailed in the results below. Low and high body fat were defined as <35% and ⩾35% total body fat for females and <25% and ⩾25% total body fat for males, respectively.

### Statistical analysis

All analyses were *a priori* stratified by sex. Histograms describing the distribution of VAT measured by DXA and MRI were constructed. VAT measured by DXA and MRI among groups stratified by race, BMI status, waist girth and percent body fat were summarized as mean (s.d.). Trends across DXA VAT quartiles were analyzed by the Jonckheere–Terpstra test for continuous variables and the Cochran–Armitage test for categorical variables. Correlation plots between DXA VAT and MRI VAT with 95% prediction limits were generated and regression equations with best-fit line and *R*^2^ with s.e. of the estimate values were calculated using ordinary least squares regression. Outliers were assessed to determine their influence on the correlation. Bland–Altman analysis using regression-based limits of agreement^19^ was performed to assess the bias and limits of agreement between DXA VAT and MRI VAT measurements. A regression-based approach was used because standard Bland–Altman analysis demonstrated heteroscedasticity and non-uniform differences across the range of measurements (that is, an increase in variability of the differences as the magnitude of the VAT measurement increased). Validation of the DXA VAT measurement using MRI VAT as the primary standard was performed using bootstrapping methods as described by Harrell *et al.*^20^ This technique may be superior to cross-validation techniques and is a recommended method for estimation of internal validity of a predictive regression model in obesity and nutrition research, as recently reviewed.^21^ Weighted kappa agreement coefficients were computed to determine the likelihood that individuals in the highest quartile of VAT by MRI would be classified in the highest quartiles of VAT by DXA. The inter-class correlation coefficient was determined by comparing DXA VAT values as measured by two independent observers for 50 randomly selected scans. Internal validity of the DXA VAT measurements was assessed by evaluating the relationship of DXA VAT quartiles with cardiometabolic risk factors. For all statistical testing, a 2-sided *P-*value <0.05 was considered statistically significant. All statistical analyses were performed using SAS version 9.4 software (SAS Institute, Cary, NC, USA).

## Results

**Figure 1:**
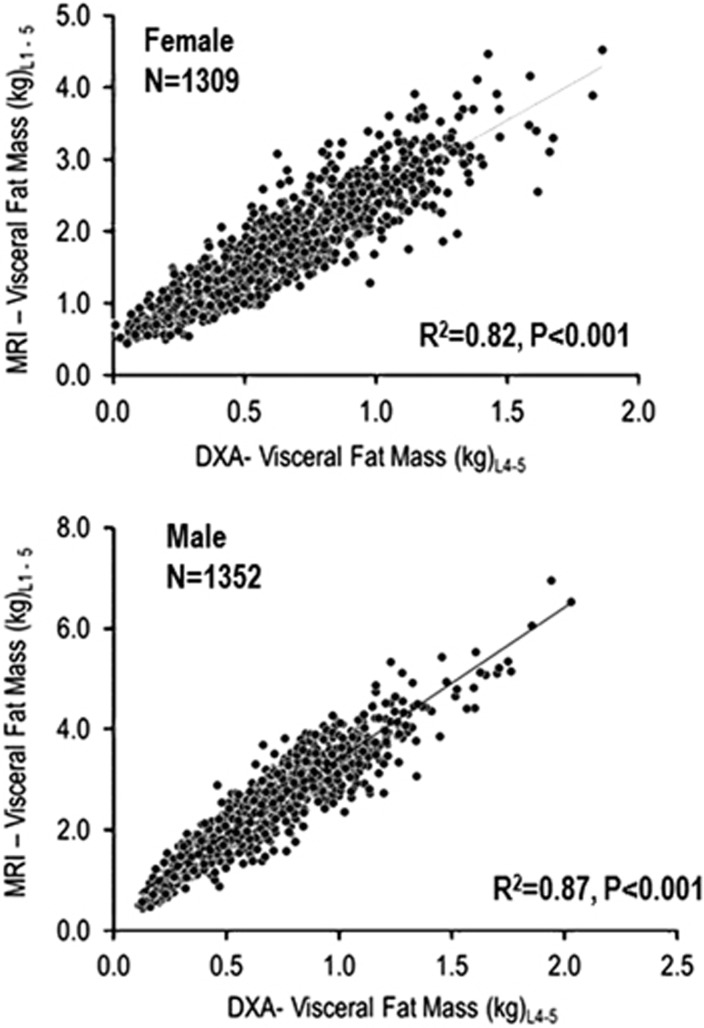
Plots of DXA VAT mass at L4–L5 vs MRI VAT mass from L1–L5 used to derive scaling factors for the estimation of total VAT mass by DXA. Plots of VAT mass estimated by DXA (kg) at intervertebral L4–L5 vs MRI-estimated VAT mass (kg) at the beginning of L1 and end of L5 vertebrae in a subgroup of non-diabetic females and males.

Table 1 describes the characteristics of the study population; mean age was 44 years, 55% were female, 48% were Black and 75% were overweight or obese. A subset of this population who did not have type 2 diabetes mellitus was selected to determine a scaling factor for conversion of DXA VAT fat mass in L4–L5 to total VAT mass in L1–L5. Plots of DXA VAT fat mass (kg) at L4–L5 vs MRI VAT mass (kg) at L1–L5 were constructed separately for females and males (Figure 1). There was a strong linear association between these two measurements (*R*^2^=0.82, *P*<0.001 for females and *R*^2^=0.87, *P*<0.001 for males). The ratio of MRI VAT mass to DXA VAT mass was calculated for each sex and racial/ethnic group (Table 2). An average scaling factor (ratio) of 2.77 was calculated for females and 3.69 for males. These factors were subsequently used to calculate the total DXA VAT fat mass of L1–L5 and the results were compared with the MRI VAT fat mass for the entire DHS population. Total VAT mass was relatively normally distributed among both females and males, with slight rightward skewness, and DXA- and MRI-measured VAT mass appeared to have similarly shaped distributions among both sexes (data not shown). The range of DXA total VAT mass was 0.03–5.22 kg among females and 0.42–7.49 kg among males.

**Figure 2:**
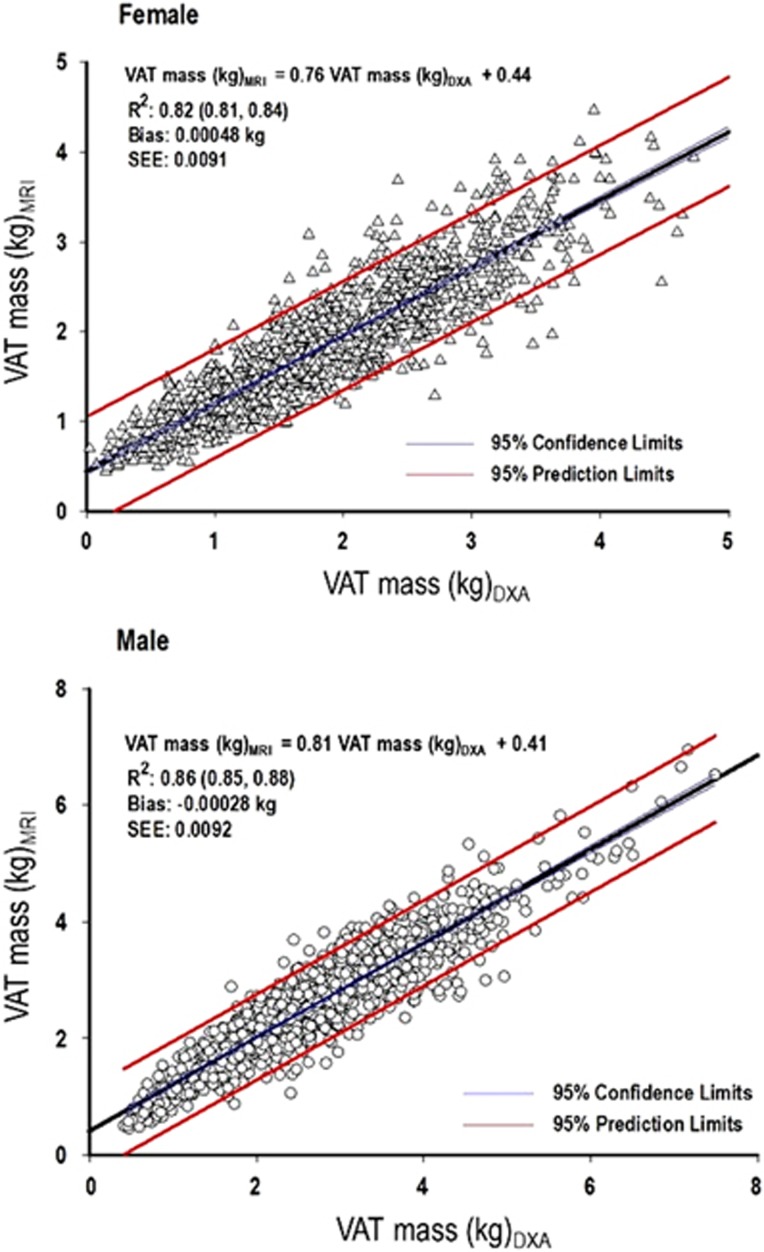
Scatterplots of VAT mass measured by DXA and MRI. Comparison of VAT mass (kg) estimated by DXA with VAT measured by MRI (kg). *R*^2^ indicates the coefficient of determination for the regression equation. 95% confidence limits of the regression line are in blue; 95% prediction limits of the correlation are in red. SEE, standard error of the estimate.

The coefficients of determination (*R*^2^) and 95% confidence limits for linear regression of MRI on DXA VAT mass stratified by sex and race are shown in Table 3 and the correlation plots with 95% limits of prediction between DXA-estimated and MRI-measured VAT mass by sex are shown in Figure 2. The *R*^2^ for linear regression of MRI on DXA VAT mass was 0.82 for females and 0.86 for males. The best-fit line describing the relationship between MRI and DXA VAT was VAT_(MRI)_ (kg)=0.76 × VAT_(DXA)_ (kg)+0.44 for females and VAT_(MRI)_ (kg)=0.81 × VAT_(DXA)_ (kg)+0.41 for males. Correlation plots between DXA-estimated and MRI-measured VAT for females and males separated by race showed similar results (Supplementary Figure S1). Quadratic and (subsequently) cubic terms were included in the regression analysis to determine whether alternative regression strategies resulted in a better fit for the relationship between DXA and MRI VAT. Both quadratic and cubic terms were not significant for females; for males, both terms were significant (*R*^2^=0.87 for quadratic and *R*^2^=0.87 for cubic, *P*<0.0001 for both), but improved negligibly on the overall model fit. Visual inspection of the scatterplots showed that the deviation from a linear relationship occurred primarily at the extremes of VAT measurement in relatively few participants (Supplementary Figure S2). Outliers analysis demonstrated that the relationship between DXA-estimated and MRI-measured VAT was insensitive to inclusion or exclusion of outliers.

**Figure 3:**
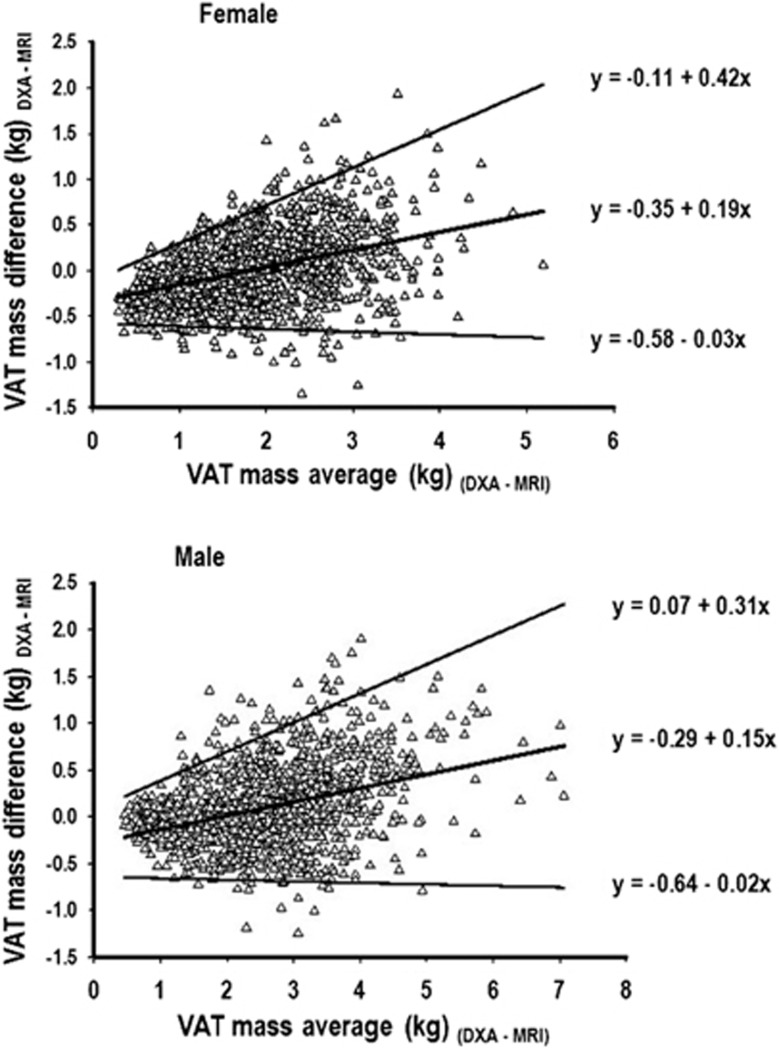
Bland–Altman analysis of agreement for VAT mass measured by DXA and MRI. Bland–Altman analysis using regression-based limits of agreement for the difference in VAT (kg) determined by DXA and MRI methods.

Mean (s.d.), mean difference and 95% limits of agreement for DXA VAT values compared with MRI VAT measurements across subgroups stratified by sex, race and waist girth are shown in Table 4. Mean differences between DXA and MRI VAT were very small in magnitude among both females and males and across racial groups. Although the magnitude of absolute differences were very small, DXA VAT appeared to slightly underestimate MRI VAT among normal weight compared with overweight/obese individuals defined by either waist girth (Table 4), BMI or total percent body fat (Supplementary Table S1), especially among females. Slight overestimation by MRI VAT was noted in the overweight/obese category, especially among males (Table 4 and Supplementary Table S1). Bland–Altman analysis characterizing the agreement between DXA and MRI VAT measurements are presented for females and males in Figure 3. In general, there was an increase in variability of the differences, as the magnitude of the VAT measurement increased. DXA tended to modestly underestimate VAT compared with MRI at lower VAT levels and overestimate it compared with MRI at higher levels. At lower VAT levels where most of the data points were centered, the regression-based limits of agreement were relatively narrow, whereas at higher VAT levels where there were less data points and more variability, the limits of agreement were wider. Similar trends were seen in the Bland–Altman analyses stratified by race for both females and males (data not shown).

Five-hundred bootstrapping repetitions were performed to determine unbiased estimates of the accuracy of the *R*^2^ resulting from the linear regression of MRI on DXA VAT. The 95% confidence interval for the *R*^2^ for females was 0.81–0.84 and for males it was 0.85–0.88, suggesting very good validity with relatively low variance in the correlation between MRI and DXA VAT. Weighted kappa agreement coefficients were computed to determine the likelihood that individuals in the highest quartile of VAT by MRI would be classified in the highest quartile of VAT by DXA. The weighted kappa coefficient and 95% confidence interval for males was 0.76 (0.74–0.78) and for females it was 0.74 (0.72–0.77), indicating substantial agreement between methods.^22^ Results were similar for each subgroup by sex and race (Supplementary Table S2). The inter-class correlation coefficient for agreement between two separate readers among 50 randomly selected scans was 0.997 (95% confidence interval 0.995–0.998) using the random effects method, demonstrating excellent inter-individual reader correlation with negligible variability. Increasing DXA VAT quartiles were positively associated with age, prevalent diabetes and metabolic syndrome, and BMI, consistent with prior observations and generally confirming internal validity of the measurement (Table 5).

## Discussion

To our knowledge, this is the largest study to date to evaluate and validate a novel DXA method of VAT quantification in a large, multiethnic cohort and the first such report to validate the DXA VAT method in comparison with MRI as the primary standard. Our results showed a strong agreement between DXA and MRI VAT across subgroups of sex, race and a wide range of BMIs, waist girth and percent body fat. DXA appeared to slightly underestimate MRI-measured VAT among normal weight individuals and overestimate MRI VAT among overweight or obese individuals, especially at very high levels of VAT. However, the mean difference between methods was 0.01 kg for females and 0.09 kg for males, both small relative to the mean VAT observed in our cohort (1.83 and 2.59 kg, respectively), and considerably less than the generally agreed upon consensus of a clinically meaningful weight difference of 5%.^23^ Further studies are needed to assess the threshold of VAT mass changes that are clinically significant in relation to cardiovascular and metabolic risk. Results were unbiasedly validated and the model was found to be well calibrated using bootstrapping techniques. Finally, inter-individual reader variability was verified to be negligible, suggesting excellent potential for the use of DXA for VAT estimation in future clinical and research applications among across a broad spectrum of populations and across multiple readers using this software.

CT or MRI are standard tools for visceral fat quantification but are suboptimal for clinical or longitudinal research use due to high radiation exposure (with CT), time-consuming image acquisition and analysis, and the need for costly, specialized equipment. DXA-based quantification of VAT has been developed as a solution and addresses many of the limitations of CT or MRI-based assessments. VAT mass measurement by DXA has been validated against CT in several smaller studies using both Hologic^11, 24^ and GE^10^ scanners. These studies reported very good agreement between the two methods with *R*^2^ values ranging between 0.865 and 0.957. However, the sample populations were relatively homogeneous (>90% white in one study and all females <49 years old in two others) and no validation against MRI-based methods was performed. The current study directly addresses these knowledge gaps and contributes a comprehensive evaluation of the applicability and validity of using DXA to estimate VAT mass for large-scale, population-based studies.

Visceral adiposity is recognized as an important risk factor for cardiometabolic disease and may be superior to anthropometric indices of obesity (such as BMI or waist circumference) for discrimination of diabetes and cardiovascular disease risk.^1, 3^ VAT mass measured by DXA has been shown to be associated with multiple cardiometabolic risk factors such as hypertension, diabetes and metabolic syndrome, independent of BMI and waist circumference in cross-sectional analyses,^25^ consistent with similar findings when VAT mass is measured by MRI.^26^ Our results confirm that DXA-derived VAT mass is positively associated with an adverse cardiometabolic profile and serve to internally validate the DXA VAT measurement in our study population. Whether DXA VAT methods may be employed for longitudinal assessment of VAT and its relation to cardiometabolic outcomes, remains to be determined. Since DXA methodology is ideal for situations requiring repeated scans over time, prospective studies including repeat measures of VAT in an observational manner or after an intervention are needed to better understand the role of DXA VAT assessment over long-term follow up.

Strengths of our study include the largest sample size for DXA VAT validation and inclusion of a cohort with large representative samples of both sexes, multiple races/ethnicities and a wide range of BMIs. Several limitations of our study also merit comment. First, we did not include participants >65 years or <30 years of age so we are unable to validate the DXA VAT method among individuals at the extremes of age. Second, since we did not repeat DXA scans with complete repositioning of the subject between scans, we are unable to measure the precision and repeatability of the DXA VAT method that may be important in assessing changes in DXA VAT longitudinally. Third, although we used robust bootstrapping techniques to validate our findings, repeat evaluation in a second large, multiethnic cohort would be ideal to fully validate the DXA VAT method for generalizable use. Fourth, as the study design is cross-sectional, we are unable to compare the accuracy of the DXA and MRI techniques for monitoring the impact of interventions targeting VAT.

## Conclusion

In conclusion, we found that a novel DXA method of VAT mass quantification was both accurate and valid compared with MRI as the primary standard among a large, multiethnic cohort of individuals with a wide range of BMIs. Our findings help to substantiate its potential use for clinical and research applications, although further studies including repeat assessments over time are needed to determine its long-term applicability.

## Figures and Tables

**Table 1:** Characteristics of the study population

*Baseline characteristics*	*Mean (s.d.*)	
	*Female*	*Male*
	(n=*1477)*	(n=*1212)*
Age (years)	44.5 (10.0)	44.3 (9.7)
		
*Race (%)*		
Black	50.2	45.6
White	30.2	34.9
Hispanic	18.3	16.3
Weight (kg)	83.3 (18.7)	86.9 (16.7)
		
*Body mass index (BMI) (kg m^−^^2^)*	30.8 (7.3)	28.4 (4.9)
BMI⩾25 (%)	76.6	74.3
		
*Waist circumference (cm)*	95.9 (16.2)	98.9 (12.8)
High waist girth^a^ (%)	66.3	39.9
Total fat mass (kg)	33.8 (12.4)	24.5 (8.9)
Total body fat (%)	41.6 (6.5)	27.9 (6.2)
Total lean mass (kg)	45.2 (8.1)	61.0 (8.9)
Hypertension (%)	31.9	28.6
Diabetes mellitus (%)	10.2	10.2
Metabolic syndrome (%)	36.6	29.2
Smokers (%)	23.7	33.2

a High waist girth defined as ⩾88 cm for females and ⩾102 cm for males.

Data are reported as mean (s.d.) or proportion (%) as appropriate.

**Table 2:** Derivation of scaling factors for estimation of total visceral fat mass by DXA

*Study group*	*Number*	*Anatomical region*		*Scaling factor (ratio)*	*Mean (s.d.) scaling factor*
		DXA L4–L5	*MRI L1–L5*		
*Visceral fat mass, kg (s.d.)*					
* Female*					
* *Black	647	0.63 (0.30)	1.72 (0.67)	2.70	2.77 (0.06)
* *White	422	0.62 (0.33)	1.76 (0.74)	2.80	
* *Hispanic	240	0.67 (0.28)	1.89 (0.67)	2.80	
					
* Male*					
* *Black	479	0.59 (0.29)	2.11 (0.91)	3.70	
* *White	398	0.76 (0.32)	2.74 (1.06)	3.61	3.69 (0.07)
* *Hispanic	172	0.72 (0.25)	2.70 (0.80)	3.75	

Abbreviations: DXA, dual-x-ray absorptiometry; MRI, magnetic resonance imaging.

**Table 3:** Coefficients of determination (*R*^2^) and 95% confidence limits for linear regression of MRI on DXA VAT mass (kg) stratified by sex and race

*Subgroup*	*Number of participants*	R^*2*^ *(95% CI)*
*Female*	*1477*	*0.82 (0.81–0.84)*
Black	741	0.79 (0.76–0.82)
White	446	0.87 (0.85–0.89)
Hispanic	270	0.82 (0.75–0.87)
		
*Male*	*1212*	*0.86 (0.85–0.88)*
Black	553	0.85 (0.82–0.88)
White	423	0.88 (0.85–0.90)
Hispanic	198	0.81 (0.74–0.86)

Abbreviations: CI, confidence interval; DXA, dual-x-ray absorptiometry; MRI, magnetic resonance imaging; VAT, visceral adipose tissue.

*R*^2^ indicates the coefficient of determination for the regression equation.

**Table 4:** Comparison of mean visceral fat mass (kg) measured by DXA and MRI

*Subgroup*	*Number of participants*	*DXA (kg) Mean (s.d.*)	*MRI (kg) Mean (s.d.*)	*Mean difference (s.d.) (kg)*	*95% Limits of agreement (kg)*
*Female*	*1477*	*1.83 (0.88)*	*1.83 (0.73)*	*0.01 (0.38)*	*−0.73–0.75*
Black	741	1.83 (0.86)	1.78 (0.70)	0.04 (0.40)	−0.74–0.82
White	446	1.77 (0.95)	1.81 (0.78)	−0.04 (0.35)	−0.73–0.65
Hispanic	270	1.98 (0.82)	1.99 (0.73)	−0.01 (0.35)	−0.70–0.68
					
*Male*	*1212*	*2.59 (1.17)*	*2.50 (1.01)*	*0.09 (0.44)*	*−0.77–0.95*
Black	553	2.29 (1.14)	2.18 (0.94)	0.11 (0.45)	−0.77–0.99
White	423	2.87 (1.23)	2.78 (1.08)	0.09 (0.42)	−0.73–0.91
Hispanic	198	2.80 (0.98)	2.77 (0.83)	0.03 (0.43)	−0.81–0.87
					
*Low waist girth*^a^					
*Female*	*498*	*1.02 (0.47)*	*1.20 (0.41)*	***−**0.18 (0.26)*	−*0.69–0.33*
Black	187	0.90 (0.41)	1.09 (0.36)	−0.19 (0.26)	−0.70–0.32
White	206	1.02 (0.47)	1.22 (0.41)	−0.20 (0.26)	−0.71–0.31
Hispanic	95	1.25 (0.48)	1.39 (0.45)	−0.15 (0.25)	−0.64–0.34
*Male*	*728*	*1.95 (0.78)*	*1.99 (0.77)*	−*0.04 (0.32)*	−*0.67–0.59*
Black	344	1.69 (0.73)	1.72 (0.70)	−0.03 (0.31)	−0.64–0.58
White	231	2.09 (0.77)	2.15 (0.78)	−0.06 (0.31)	−0.67–0.55
Hispanic	125	2.33 (0.75)	2.42 (0.71)	−0.09 (0.35)	−0.78–0.60
					
*High waist girth*^a^					
*Female*	*979*	*2.25 (0.74)*	*2.15 (0.65)*	*0.10 (0.39)*	−*0.66–0.86*
Black	554	2.14 (0.73)	2.02 (0.62)	0.12 (0.41)	−0.68–0.92
White	240	2.42 (0.76)	2.32 (0.66)	0.10 (0.37)	−0.63–0.83
Hispanic	175	2.38 (0.71)	2.31 (0.64)	0.07 (0.39)	−0.69–0.83
*Male*	*484*	*3.55 (0.98)*	*3.26 (0.84)*	*0.30 (0.51)*	−*0.70–1.30*
Black	209	3.28 (0.97)	2.94 (0.77)	0.34 (0.54)	−0.72–1.40
White	192	3.81 (0.99)	3.54 (0.87)	0.27 (0.47)	−0.65–1.19
Hispanic	73	3.61 (0.78)	3.38 (0.67)	0.22 (0.49)	−0.74–1.18

Abbreviations: DXA, dual-x-ray absorptiometry; MRI, magnetic resonance imaging.

a Low waist girth for females <88 cm and for males <102 cm; high waist girth for females ⩾88 cm and for males ⩾102 cm.

**Table 5:** Clinical and laboratory characteristics by DXA VAT mass quartiles

*Baseline characteristics*	*Female*				*Male*			
	*Q1*	*Q2*	*Q3*	*Q4*	*Q1*	*Q2*	*Q3*	*Q4*
	(n=*369)*	(n=*370)*	(n=*369)*	(n=*369)*	(n=*303)*	(n=*303)*	(n=*303)*	(n=*302)*
DXA VAT mass range (kg)	0.03–1.17	1.18–1.76	1.77–2.46	2.47–5.22	0.42–1.73	1.74–2.49	2.50–3.32	3.33–7.49
Age (years)	40.0 (9.5)	43.6 (9.3)^a^	45.8 (9.8)^a^	48.3 (9.5)^a^	41.2 (9.5)	43.1 (9.5)^a^	44.6 (8.9)^a^	48.4 (9.4)^a^
								
*Race (%)*								
Black	48.2	52.7^a^	50.9^a^	48.8^a^	64.7	46.2^a^	39.6^a^	32.0^a^
White	39	25.4	26.6	29.8	26.1	30.7	36.9	45.9
Hispanic	10.6	21.3	20.6	20.6	7.6	18.8	19.8	19.1
Body mass index (kg m^−^^2^)	23.9 (4.3)	28.8 (4.7)^a^	32.6 (5.8)^a^	37.8 (6.1)^a^	23.7 (3.2)	27.2 (3.5)^a^	29.8 (3.4)^a^	32.9 (3.8)^a^
Hypertension (%)	14.4	25.4^a^	38.2^a^	49.6^a^	11.9	22.4^a^	33.3^a^	46.9^a^
Diabetes (%)	2.2	4.9^a^	11.9^a^	21.9^a^	3.9	7.3^a^	10.9^a^	18.8^a^
Metabolic syndrome (%)	5.7	25.4^a^	47.2^a^	68.3^a^	3.6	14.9^a^	34.6^a^	63.7^a^
DXA fat mass (kg)	21.7 (7.4)	30.7 (7.5)^a^	37.1 (9.5)^a^	45.7 (10.1)^a^	14.9 (4.9)	22.3 (5.2)^a^	27.1 (5.7)^a^	33.5 (7.2)^a^
DXA body fat (%)	34.4 (5.9)	41.1 (4.1)^a^	44.1 (4.0)^a^	46.8 (4.2)^a^	20.7 (4.7)	27.2 (3.8)^a^	30.1 (3.9)^a^	33.9 (3.8)^a^
DXA lean mass (kg)	40.0 (5.9)	43.4 (6.5)^a^	46.3 (7.4)^a^	51.3 (7.7)^a^	56.1 (7.5)	59.5 (8.5)^a^	62.4 (8.0)^a^	66.1 (8.5)^a^
MRI VAT mass (kg)	1.02 (0.28)	1.56 (0.30)^a^	2.02 (0.37)^a^	2.72 (0.53)^a^	1.31 (0.42)	2.17 (0.39)^a^	2.81 (0.45)^a^	3.69 (0.73)^a^

Abbreviations: DXA, dual-x-ray absorptiometry; VAT, visceral adipose tissue.

a *P*<0.0001; *P*-trend analyzed by Jonckheere–Terpstra test for continuous variables and Cochran–Armitage test for categorical variables.

Q, quartile data are reported as mean (s.d.) or proportion (%) as appropriate.
