# Supplementary material for: Comparison of visceral fat mass measurement by dual-X-ray absorptiometry and magnetic resonance imaging in a multiethnic cohort: the Dallas Heart Study
Source: Nutr Diabetes. 2016 Jul 18;6(7):e221–. doi: 10.1038/nutd.2016.28 (PMC4973141; doi:10.1038/nutd.2016.28)

**SUPPLEMENTAL MATERIAL**

**Table S1.** Comparison of Mean Visceral Fat Mass (kg) Measured by DXA and MRI Stratified by BMI and Percent Body Fat Sex-Specific Cut Points

| **Subgroup** | **Sample Size** | | **DXA (kg)**  **Mean (SD)** | **MRI (kg)**  **Mean (SD)** | **Mean Difference (SD)**  **(kg)** | **95% Limits of Agreement**  **(kg)** |  |
| --- | --- | --- | --- | --- | --- | --- | --- |
| **BMI<25** |  |  |  |  |  |  |  |
| **Female** |  | **345** | **0.92 (0.48)** | **1.13 (0.43)** | **-0.21 (0.25)** | **-0.70 to 0.28** |  |
| Black |  | 126 | 0.84 (0.45) | 1.03 (0.37) | -0.18 (0.27) | -0.71 to 0.35 |  |
| White |  | 160 | 0.97 (0.49) | 1.19 (0.45) | -0.22 (0.25) | -0.71 to 0.27 |  |
| Hispanic |  | 50 | 0.97 (0.42) | 1.19 (0.43) | -0.22 (0.19) | -0.59 to 0.15 |  |
| **Male** |  | **312** | **1.47 (0.66)** | **1.53 (0.65)** | **-0.06 (0.25)** | **-0.55 to 0.43** |  |
| Black |  | 163 | 1.30 (0.61) | 1.36 (0.62) | -0.07 (0.22) | -0.50 to 0.36 |  |
| White |  | 104 | 1.62 (0.69) | 1.68 (0.69) | -0.06 (0.26) | -0.57 to 0.45 |  |
| Hispanic |  | 32 | 1.69 (0.56) | 1.79 (0.49) | -0.10 (0.34) | -0.77 to 0.57 |  |
| **BMI≥25** | |  |  |  |  |  |  |
| **Female** | | **1132** | **2.11 (0.78)** | **2.04 (0.67)** | **0.07 (0.38)** | **-0.67 to 0.81** |  |
| Black | | 615 | 2.03 (0.78) | 1.94 (0.64) | 0.09 (0.40) | -0.69 to 0.87 |  |
| White | | 286 | 2.23 (0.83) | 2.16 (0.71) | 0.06 (0.36) | -0.65 to 0.77 |  |
| Hispanic | | 220 | 2.21 (0.71) | 2.17 (0.66) | 0.04 (0.36) | -0.67 to 0.75 |  |
| **Male** | | **900** | **2.97 (1.05)** | **2.83 (0.90)** | **0.14 (0.47)** | **-0.78 to 1.06** |  |
| Black | | 390 | 2.71 (1.04) | 2.52 (0.84) | 0.19 (0.50) | -0.79 to 1.17 |  |
| White | | 319 | 3.28 (1.08) | 3.14 (0.93) | 0.14 (0.46) | -0.76 to 1.04 |  |
| Hispanic | | 166 | 3.02 (0.89) | 2.96 (0.75) | 0.05 (0.44) | -0.81 to 0.91 |  |
|  | |  |  |  |  |  |  |
| **Low Body Fat*** | |  |  |  |  |  |  |
| **Female** | | **220** | **0.72 (0.38)** | **0.99 (0.36)** | **-0.27 (0.21)** | **-0.68 to 0.14** |  |
| Black | | 103 | 0.74 (0.41) | 0.99 (0.39) | -0.25 (0.20) | -0.64 to 0.14 |  |
| White | | 81 | 0.68 (0.35) | 0.97 (0.32) | -0.29 (0.23) | -0.74 to 0.16 |  |
| Hispanic | | 28 | 0.80 (0.37) | 1.07 (0.39) | -0.27 (0.15) | -0.56 to 0.02 |  |
| **Male** | | **344** | **1.44 (0.61)** | **1.53 (0.63)** | **-0.09 (0.27)** | **-0.62 to 0.44** |  |
| Black | | 214 | 1.35 (0.58) | 1.43 (0.59) | -0.08 (0.28) | -0.63 to 0.47 |  |
| White | | 90 | 1.51 (0.65) | 1.60 (0.66) | -0.09 (0.23) | -0.54 to 0.36 |  |
| Hispanic | | 35 | 1.76 (0.60) | 1.98 (0.64) | -0.21 (0.25) | -0.70 to 0.28 |  |
|  | |  |  |  |  |  |  |
| **High Body Fat*** | |  |  |  |  |  |  |
| **Female** | | **1257** | **2.03 (0.79)** | **1.98 (0.68)** | **0.05 (0.38)** | **-0.69 to 0.79** |  |
| Black | | 638 | 2.00 (0.77) | 1.91 (0.65) | 0.09 (0.40) | -0.69 to 0.87 |  |
| White | | 365 | 2.02 (0.86) | 2.00 (0.73) | 0.02 (0.35) | -0.67 to 0.71 |  |
| Hispanic | | 242 | 2.11 (0.75) | 2.10 (0.68) | 0.02 (0.35) | -0.67 to 0.71 |  |
| **Male** | | **868** | **3.04 (1.02)** | **2.88 (0.87)** | **0.16 (0.47)** | **-0.76 to 1.08** |  |
| Black | | 339 | 2.88 (0.99) | 2.65 (0.80) | 0.23 (0.50) | -0.75 to 1.21 |  |
| White | | 333 | 3.24 (1.08) | 3.10 (0.94) | 0.14 (0.45) | -0.74 to 1.02 |  |
| Hispanic | | 163 | 3.02 (0.90) | 2.94 (0.77) | 0.08 (0.45) | -0.80 to 0.96 |  |
| *Low body fat <35% for females and <25% for males; high body fat ≥35% for females and ≥25% for males | | | | | |  |  |

**Table S2.** Weighted Kappa Coefficient for Agreement of DXA Quartile 4 and MRI Quartile 4 for VAT mass by Sex and Race

| **Subgroup** | **Number of Participants** | | **Weighted Kappa (95% CI)** |
| --- | --- | --- | --- |
| **Female** |  | **369** | **0.74 (0.72-0.77)** |
| Black |  | 185 | 0.72 (0.69-0.75) |
| White |  | 111 | 0.78 (0.75-0.82) |
| Hispanic |  | 67 | 0.69 (0.64-0.75) |
| **Male** |  | **303** | **0.76 (0.74-0.78)** |
| Black |  | 138 | 0.77 (0.73-0.80) |
| White |  | 106 | 0.77 (0.73-0.81) |
| Hispanic |  | 49 | 0.74 (0.68-0.80) |
| CI=confidence interval | | | |

**/Figure S1.** Scatterplots of VAT mass (kg) measured by DXA and by MRI stratified by Sex and Race


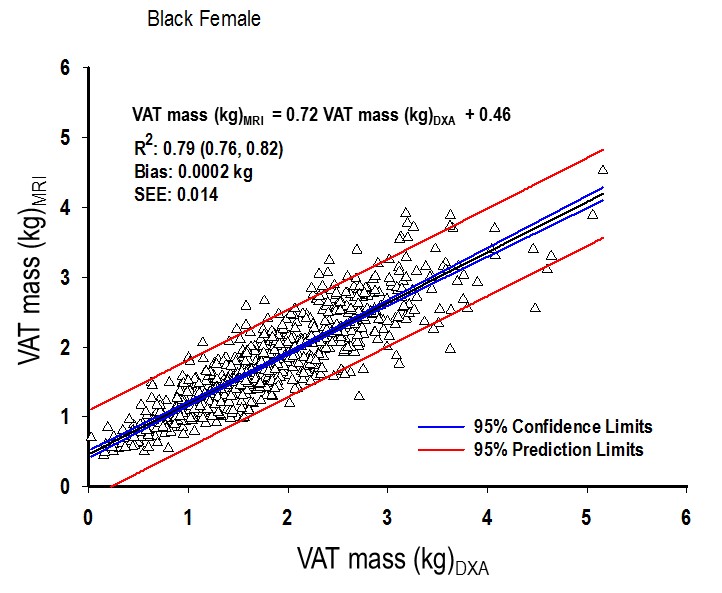


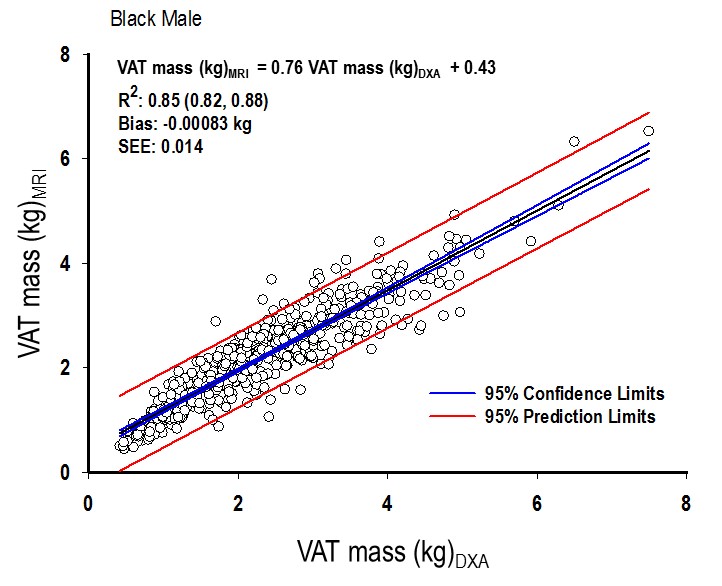


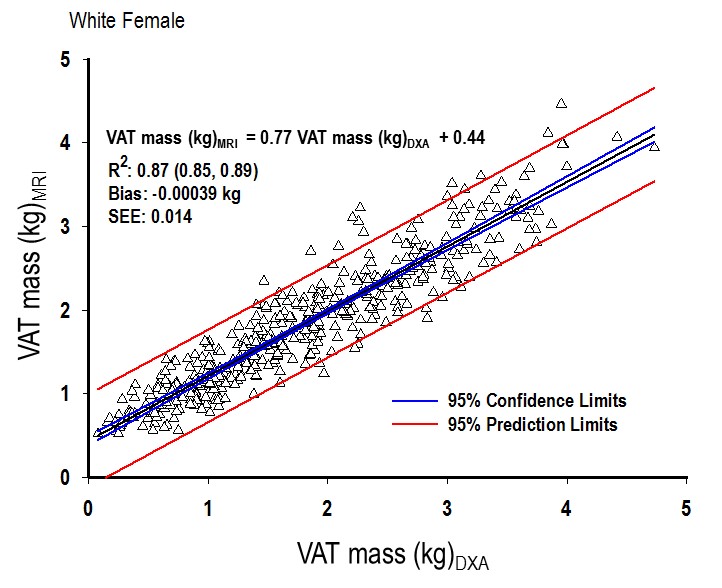


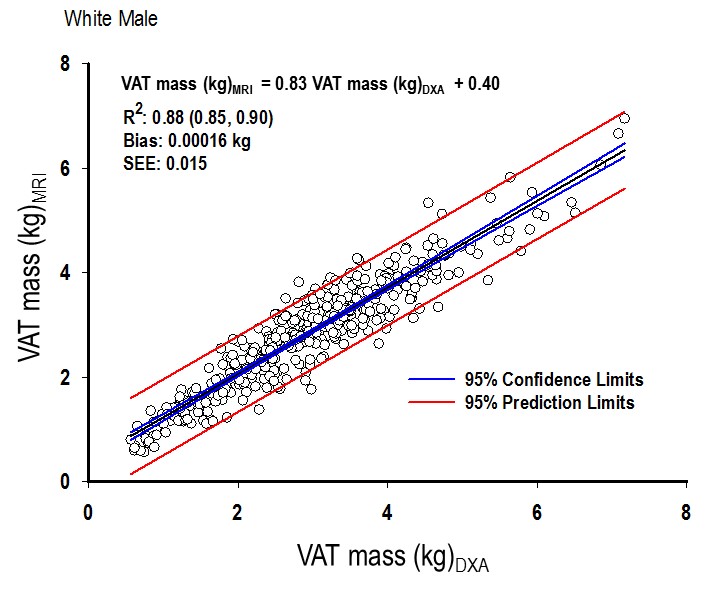


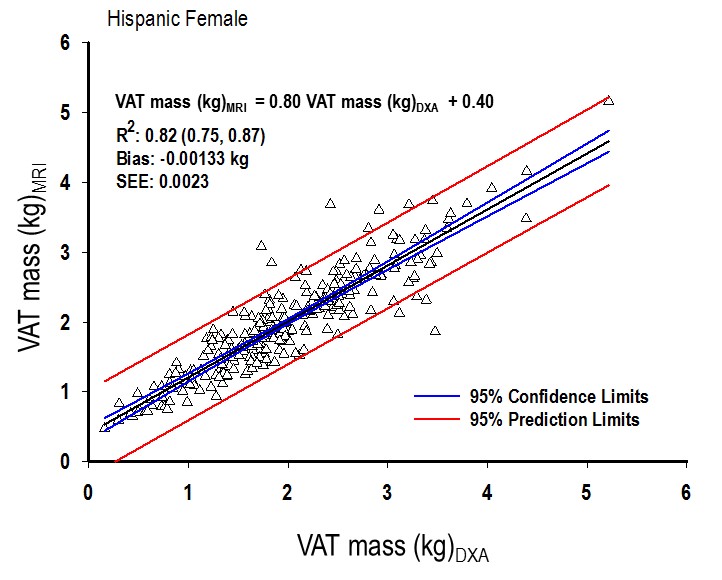


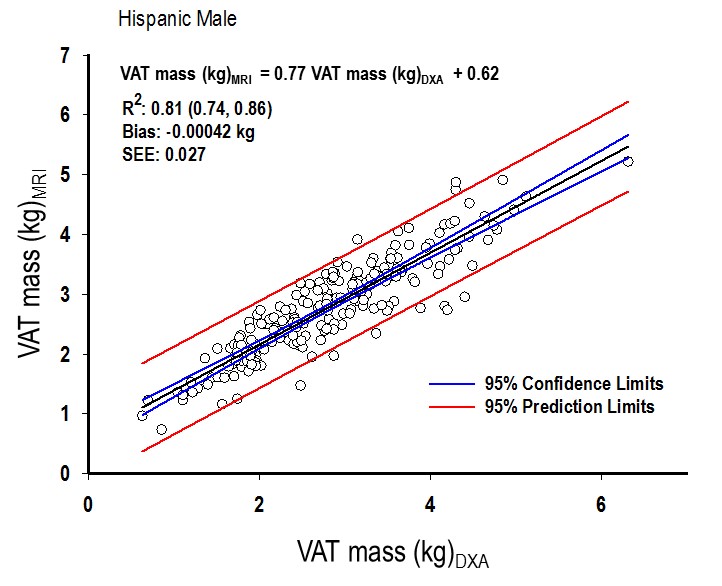


**Figure S2.** Sex-stratified scatterplots to assess alternative regression models fit for DXA – MRI VAT mass correlation


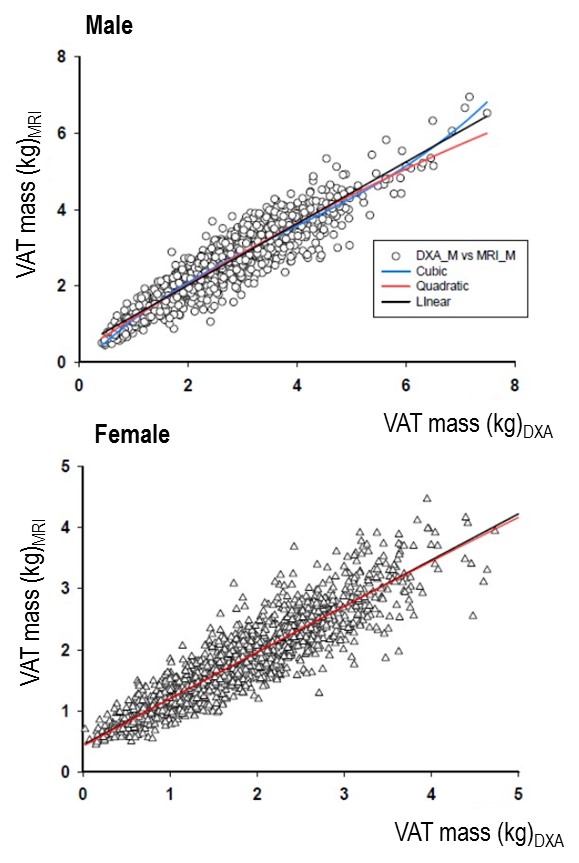

Supplement: Supplementary Information [file nutd201628x1.doc]
